# Principles for Chronic Pain Management in the Adult Traumatic Spinal Cord Injury Population at the Primary Healthcare Level, in a Developing Context: A Delphi Study

**DOI:** 10.1177/27536130251349456

**Published:** 2025-07-07

**Authors:** Tammy-Lee Williams, Lena Nilsson Wikmar, Conran Joseph

**Affiliations:** 1 Department of Physiotherapy, Faculty of Community and Health Sciences, 108325 University of the Western Cape, Cape Town, South Africa; 2 Department of Neurobiology, Faculty of Care Sciences and Society, 27106 Karolinska Institutet, Stockholm, Sweden; 3 Division of Physiotherapy, Faculty of Health and Rehabilitation services, 26697 Stellenbosch University, Stellenbosch, South Africa

**Keywords:** chronic pain, traumatic spinal cord injury, management principles, treatment principles, holistic, biopsychosocial, primary healthcare, Western Cape, South Africa

## Abstract

**Introduction:**

Individuals with traumatic spinal cord injury (TSCI) are dissatisfied with their chronic pain management. A biopsychosocial approach has been proven to improve chronic pain. Guidelines are required to holistically manage chronic pain in the TSCI population.

**Methods:**

A Delphi study was conducted to gain consensus on design principles for chronic pain in the TSCI population, for the Western Cape of South Africa. Purposive sampling was used to recruit first-line primary health care providers from primary health care settings in the Cape Metropolitan region. Participants were asked for consent on principles pertaining to the assessment, education and planning for chronic pain management, pharmacological and non-pharmacological therapy for neuropathic and nociceptive pain, as well as the monitoring of chronic pain and referral of resistant pain. For consensus analysis, a median of 3.24 or higher was considered in addition to two categories of consensus, namely weak consensus (50%–70%) and strong consensus (>70%).

**Results:**

The first-line primary health care providers agreed on eighteen principles to guide chronic pain management in the TSCI population. Consensus could not be reached on the second to fourth line pharmacological management of neuropathic pain.

**Conclusion:**

The agreed upon design principles may be considered as starting points for implementation at the primary health care level in the Western Cape of South Africa.

## Introduction

Chronic pain has high prevalence rates in the TSCI population, surfacing within the first few years following the traumatic incident.^1-3^ Chronic pain in this study population consists of nociceptive pain (NP) and neuropathic pain (NeuP).

The focus of chronic pain management in the TSCI population, in South Africa leans towards a pharmacological approach, which may be evident in two studies depicting opioid misuse amongst TSCI survivors and individuals expressing the lack of non-pharmacological referrals from the primary health care setting.^4,5^ Persons with TSCI (PWTSCI), locally and abroad, have voiced their dissatisfaction with the current medical approach to chronic pain management, accentuating its temporary pain relief and undesirable side-effects.^5-8^

A recent study has demonstrated the physiotherapist use of non-pharmacological management strategies for chronic pain in the SCI population and these include cognitive behavioural therapy, electrotherapy/TENS, desensitisation, graded activity, joint mobilisation, myofascial release pain neuroscience education, exercises, neural tissue mobilisation and self-management techniques.^9^ The efficacy of these management strategies amongst various types of chronic pain are demonstrated in the literature.^10-13^ However, its consideration for use in the local setting remains unknown.

Various factors may influence the optimal management of chronic pain in general and in the SCI population. These include poor knowledge and frustrations by health care providers, disregarding the patient’s perspective, affordability of care, time constraints on health care providers and lack of support for patients.^14-17^ One study reported that health care providers expressed their lack of knowledge and expertise in managing chronic pain.^14^ Furthermore, the patient-healthcare provider relationship is essential to the optimal care of chronic pain. Health care providers have highlighted the importance of developing this relationship into one of mutual understanding and goal planning.^15,16^ Further challenges to optimal chronic pain management specifically in the SCI population include affordability of care, particularly for non-pharmacological therapies; time constraints which limit health care providers ability to adequately educate patients on chronic pain and the management thereof.^17^

A holistic approach to chronic pain management had been suggested as early as 1977, with George Engel^18^ introducing the biopsychosocial model to pain management. More recently, the World Health Organization (WHO) has advocated for interprofessional and collaborative practice in order to address the complex needs of patients.^19^ In South Africa, challenges exist which influence the holistic management of care. These include, but are not limited to staff shortages, inadequate resources, time constraints, ineffective referral system and poor knowledge by health care providers of holistic management.^20,21^ Therefore, high-quality guidelines are needed to adopt a systematic biopsychosocial approach to chronic pain management,^22^ particularly for the TSCI population and in the South African context. In a recent scoping review, guidelines for chronic pain in the SCI population, in Germany, were identified and critically appraised.^23^ The guideline was appraised as a high-quality guideline according to the AGREEII tool. The guideline includes recommendations for assessment and classification of chronic pain and its sub-types; pharmacological and non-pharmacological recommendations for NP and NeuP as well as the recommendations for the monitoring of chronic pain and management of resistant chronic pain. Contextualisation of these guidelines for the South African context is required, as a developed country (Germany) will make provision for a larger distribution of resources such as professional services and medication, etc.

## Methodology

### Research Design

Developed in 1963^24^, the Delphi study technique aims to gather and combine opinions from participants on a certain topic, based on their experience. The process should be repeated until consensus is reached or until it becomes clear that no consensus is possible.^25^ In this study, a modified Delphi technique was used to gather health care provider opinions and consensus on a set of principles which should be included, at the primary health care level, to guide the management of chronic pain amongst the TSCI population. The traditional technique consists of a first round of idea/theme construction, a second round of consensus building and a final round of prioritisation.^26^ The authors made use of a modified Delphi study as an initial round of idea/theme construction was not required since a list of guiding principles was already sought through a scoping review and rated with good methodological rigor as per the AGREEII tool,^27^ as well as themes from previous qualitative interviews conducted with health care providers at the primary health care level^28^ and PWTSCI,^5^ in the Cape Metropolitan region.

### Population and Sampling

Purposive sampling was used to recruit nurses and doctors (medical officers) knowledgeable about chronic pain management at the primary health care level. Inclusion criteria included a minimum of two years’ professional experience and currently working at the primary health care level of the public sector.

### Setting

Primary health care clinics across the Cape metropolitan region (of the Western Cape) was chosen as the research setting. Primary health care is concerned with essential, evidence-based, affordable, ethical and equitable care provided at a community level,^29^ and is the firs-line of care for many people in developing countries. In the Western Cape, 75% of its population depend on public-sector health facilities, especially for chronic conditions.^30^ Unfortunately, at the primary health care level in the Western Cape, no specialized pain centres exist.^31^

### Data Collection

Fourteen City of Cape Town clinics were identified as primary health care settings who attended to PWTSCI. The managers of the health care clinics were emailed to disseminate the invitation to participate, which included the approval letter to conduct the study in the clinic (Reference 1094, from the City of Cape Town, City Health), the ethics approval from *the* University of the Western Cape’s Biomedical Research and Ethics Committee (Reference 20_8_22), the Google form® which contained the questionnaire, an information sheet as well as the consent form. In addition, in-person recruitment at the various clinics as well as snowballing recruitment techniques were employed.

**Round 1:** The health care providers were asked to rate each principle on a 5-point Likert scale (from strongly disagree to strongly agree), for inclusion in a set of principles guiding chronic pain management in the TSCI population within the local Western Cape, of South Africa, region. The principles related to the assessment and education of chronic pain as well as the planning for the management of chronic pain, such as setting realistic goals and including the patient in the decision-making. Participants were also asked to provide comments at the end of the questionnaire to include additional principles which may have been omitted and/or provide general comments. In addition, the participants were asked to provide their email addresses for the follow-up Delphi study rounds. Round one consisted of emailing the managers of the clinics. Following poor response via email, the researcher employed in-person recruitment, which involved the researcher attending the various clinics to recruit first-line primary health care providers for the study. Prospective participants were given information regarding the purpose of the study and were provided the opportunity to voluntarily participate. Furthermore, snowball recruitment (recruitment of participants by health care providers already involved in the study) was also employed, which resulted in the participation of 28 first-line primary health care providers.

**Round 2:** In round 2, the first-line primary health care providers were contacted via the email addresses provided and asked to decide which class of medication was best suited for each line of pharmacological therapy for neuropathic pain. Following poor response via email, the researcher employed in-person recruitment which resulted in the participation of ten first-line primary health care providers. Most of the participants were from the initial cohort and a few were new participants. Professionals were asked to rate principles regarding the monitoring of chronic pain as well as principles regarding the management of resistant pain, on a 5-point Likert scale (from strongly disagree to strongly agree).

### Data Analysis

In the second round, data coding took place in SPSS V29, for the various pharmacological drugs: Neuropathic pain, opiate (narcotic) analgesic was labelled “1”, anticonvulsants were labelled “2”, tricyclic antidepressants were labelled “3”, Selective serotonin norepinephrine reuptake inhibitors (SNNRIs) were labelled “5” and for nociceptive pain, opioid analgesics were labelled “4” and non-opioid analgesics were labelled “6”. In the first and second rounds the responses from the Likert scale was divided into three categories, with “agree” and “strongly agree” comprising the consensus category; “disagree” and “strongly disagree” comprising the non-consensus category and lastly, “neutral” comprising the unsure category. The consensus category was further divided into weak consensus (50%–70%) and strong consensus (>70%).^25,32^ Furthermore, a median of 3.24 or higher was also considered as it more appropriately displays consensus amongst a panel, as discussed by Hsu and Sandford.^25^

### Ethics

Permission to conduct the study at the identified clinics was obtained from the City of Cape Town City Health (Reference 1094). Ethics approval to conduct the study was obtained from The University of the Western Cape’s Biomedical Research and Ethics Committee (Reference 20_8_22). Participants provided informed consent prior to completing the questionnaires.

## Results

### Participant Characteristics

Twenty-eight participants took part in round one of the Delphi study. Only ten professionals completed the second round of the Delphi study. The results of the Delphi study have therefore been displayed according to each round. Most of the participants were female (round one, n *= 28* and round two, n *= 9*). The ages of the participants ranged from range 27-55 years (round one, *SD* = 7.9 and round two, *SD* = 6.7) and their years of experience ranged from range 2-35 years. The cohort included nursing clinical managers, registered nurse, clinical nurse practitioner, enrolled nurse, enrolled nursing assistant and professional nurse and medical officers (doctors).

### Main Findings

The participants reached strong consensus (>70%) on most of the guidelines. Weak consensus was attained on two guidelines pertaining to the classification of chronic pain as well as the first-line pharmacological management of neuropathic pain.

### Assessment, Education and Planning in Relation to Chronic Pain Management

As seen in Table 1, strong consensus was attained regarding all aspects relating to the assessment, education of and planning for chronic pain.

**Table 1.** Assessment, Education and Planning Principles for chronic Pain Management in the TSCI Population Through consensus in Round One.

Guideline	Consensus	Median (range)
Classify the pain using the international spinal cord injury pain (ISCIP) classification or the international association for the study of pain (IASP) classification	67.9% (weak)	4.0 (3-5)
Differentiate between traumatic spinal cord injury (TSCI) and non-traumatic spinal cord injury (NTSCI)	100% (strong)	4.5 (4-5)
Eliminate other causes of pain, such as other pathologies and comorbidities common in the TSCI population (for example, bladder infection, bladder stones, kidney infections, heterotrophic ossification, bowel impaction, hypertension, mental illness etc.)	96.4% (strong)	4.0 (3-5)
Assess psychosocial factors which could contribute to pain / referral to social worker and psychologist for further assessment	92.9% (strong)	5.0 (2-5)
Assess physical factors such as positioning in wheelchair, ill-fitting clothing etc. / referral to the physiotherapist for further assessment	96.4% (strong)	5.0 (3-5)
Rate the severity of the pain using a pain rating scale, together with the aggravating and easing factors	96.4% (strong)	5.0 (3-5)
Assess past management such as medications prescribed and previous allied health (physiotherapist, psychologist, social worker, occupational therapist etc.) attendance / recommendations	96.4% (strong)	4.0 (3-5)
Education of client and family members regarding pain physiology, presentation, self-management and monitoring of pain	100% (strong)	5.0 (4-5)
Set realistic goals and include client in decision-making regarding pain management	96.4% (strong)	5.0 (3-5)
Chronic pain should be managed with a multidisciplinary approach, that is, pharmacological management combined with physiotherapy, occupational therapy, social worker intervention, psychotherapy, dietician etc.	96.4% (strong)	5.0 (4-5)

### Pharmacological Management of Chronic Pain

Consensus regarding the pharmacological management of nociceptive pain was obtained in the first round of the study. Regarding neuropathic pain, many participants were unsure regarding the specific drug name in relation to the line of therapy. Therefore, the second round of the Delphi study asked participants to recommend the class of drugs appropriate for the line of therapy, for neuropathic pain. However, as seen in the first round, the participants could only agree on Tricyclic antidepressants (TCA) as first-line therapy for neuropathic pain. A few of the participants commented that many of the drug names listed were not available at the primary health care level and certain drugs could only be prescribed by specialists and not at the primary health care level. No consensus could be reached on the recommendation for second to fourth-line pharmacological therapy, for neuropathic pain. The second-line pharmacotherapy for neuropathic pain was listed as Duloxetine [selective serotonin and norepinephrine reuptake inhibitors (SSNRIs)], thereafter, Amitriptyline [tricyclic antidepressant (TCA)]. The third-line was listed as Oxycodone [opiate (narcotic) analgesics], thereafter Lamotrigine [anticonvulsant]. The fourth-line was listed as Venlafaxine [selective serotonin and norepinephrine reuptake inhibitors (SNRIs)] and thereafter Opioid analgesics: Tramadol.

The consensus results can be viewed in Table 2.

**Table 2.** Principles for the Pharmacological Management of chronic Pain in the TSCI Population, Through consensus in Round One and Two.

Principle	Consensus	Median (Range)
Round One		
First-line pharmacological therapy for nociceptive pain: Non-opioid analgesics	90% (strong)	6.0 (4-6)
Second-line pharmacological therapy for nociceptive pain: Opioid analgesics	70% (strong)	4.0 (4-6)
Round two		
First-line pharmacological therapy for neuropathic pain: Tricyclic antidepressants	50% (weak)	3.5 (3-5)

### Non-pharmacological Management of Chronic Pain

Table 3 describes the strong consensus regarding the non-pharmacological management of chronic pain.

**Table 3.** Principles for the Non-pharmacological Management of chronic Pain in the TSCI Population, Through consensus in Round One.

Principle	Consensus	Median (range)
Round one		
First-line non-pharmacological therapy for nociceptive pain: Physiotherapeutic interventions, physical activity, exercise	71.4% (strong)	4.0 (2-5)
Second-line non-pharmacological therapy for neuropathic pain: Physiotherapeutic measures and physical activity, exercise, TENS, massage, heat therapy. Psychotherapy (hypnotherapy, cognitive-behavioural therapy). Occupational therapy, social worker intervention, dietetics	85.7% (strong)	4.0 (3-5)

### Additional Comments on the Management of Chronic Pain

The first-line primary health care providers were given an opportunity to comment on any additional principles that should be considered in the context of the Western Cape. One participant mentioned that interprofessional approaches to manage chronic pain is problematic as staff scarcity remains an issue at the primary health care level. Furthermore, the participants also commented that at the primary health care level, the only drugs which are available include amitriptyline, paracetamol, ibuprofen and tramadol. Drugs such as pregabalin and gabapentin are only available at the specialist level of care.

### Monitoring of Chronic Pain Management and Management of Resistant Chronic Pain

Table 4 indicates that strong consensus existed for the principles relating to the monitoring of chronic pain and management of resistant chronic pain.

**Table 4.** Principles for the Monitoring of chronic Pain and Management of Resistant chronic Pain, Through consensus in Round 2.

Principle	Consensus	Median (range)
Monitor pain using the same pain scale as used with initial assessment, combined with re-evaluation of aggravating and easing factors	100% (strong)	4.0 (4-5)
With insufficient neuropathic pain relief with pharmacotherapy, consider combining pharmacological therapy	90% (strong)	4.0 (3-5)
For persistent insufficient pain relief, consider referral to a pain clinic	80% (strong)	4.0 (2-5)

**Figure 1.**
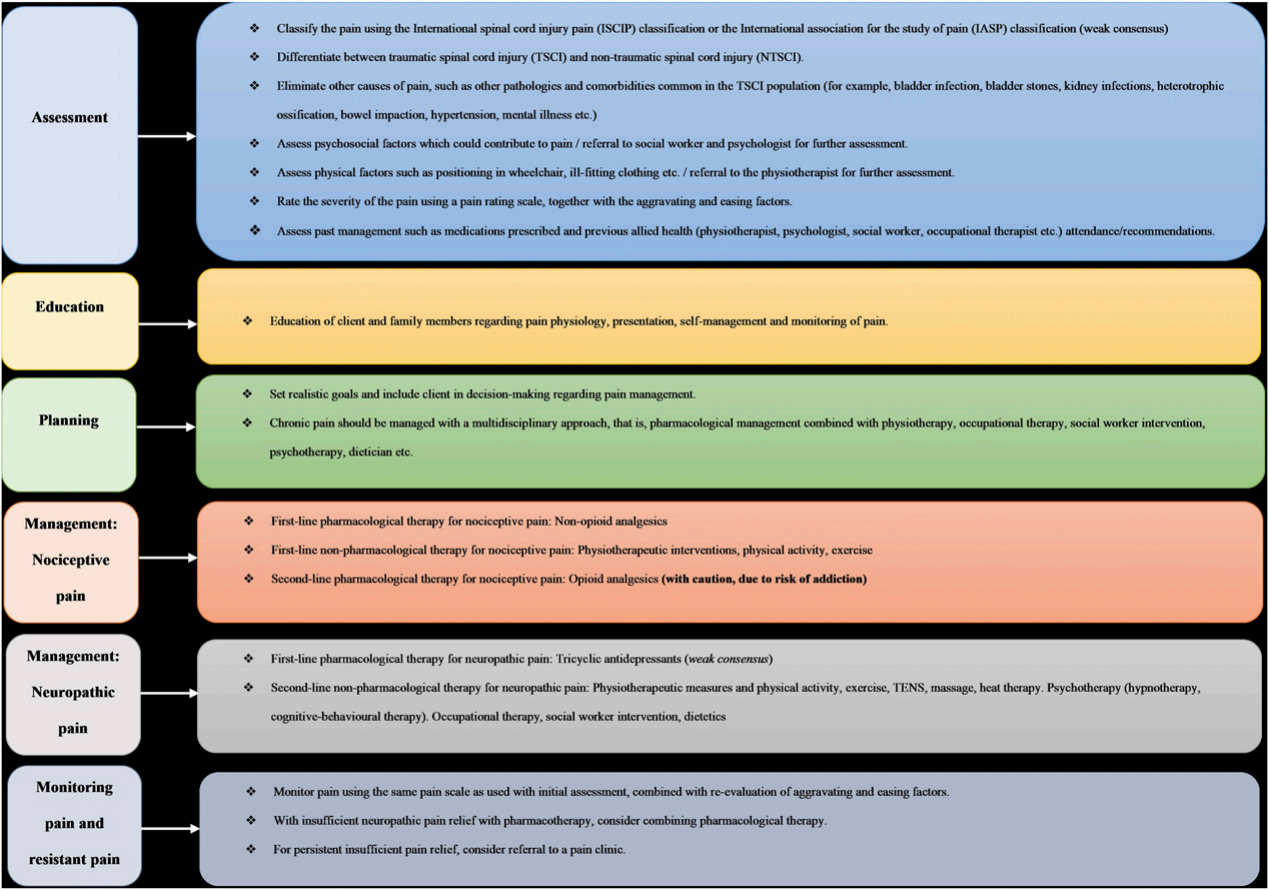
Design principles for chronic pain management in the TSCI population, in South Africa. The blue section displays the assessment principles for chronic pain. The yellow section indicates principles relating to the education of chronic pain and its management. The green section displays the planning involved in the management of chronic pain. The orange indicates the treatment options for the management of nociceptive pain. The grey displays the treatment options for the management of neuropathic pain. The final block illustrates the principles for monitoring chronic pain as well as management of resistant chronic pain.

Figure 1 illustrates the final compilation of design principles for chronic pain in the TSCI population, which gained consensus in this Delphi study.

## Discussion

The aim of the study sought to gain consensus on principles to guide the management of chronic pain in the TSCI population, in the developing context of the Western Cape of South Africa. The principles were based on themes explored in qualitative papers from patients^5^ and health care providers’ perspectives.^28^ In addition to published German guidelines for chronic pain in the TSCI population, retrieved and assessed with the AGREEII tool by the authors.^27^ The first-line primary health care providers agreed on 18 principles pertaining to the assessment of chronic pain. These included the first-line pharmacological management for neuropathic and nociceptive pain, first-line non-pharmacological therapy for nociceptive pain, second-line non-pharmacological therapy for neuropathic pain, second-line pharmacological therapy for nociceptive pain as well as three principles in relation to the monitoring of chronic pain and the management of resistant pain. No consensus could be reached on the appropriate recommendation for the second- to fourth-line pharmacological management of neuropathic pain, which included Duloxetine [selective serotonin and norepinephrine reuptake inhibitors (SSNRIs)], thereafter, Amitriptyline [tricyclic antidepressant (TCA)] (second-line); Oxycodone [opiate (narcotic) analgesics], thereafter Lamotrigine [anticonvulsant] (third-line), and Venlafaxine [selective serotonin and norepinephrine reuptake inhibitors (SNRIs)], thereafter Opioid analgesics: Tramadol (fourth-line).

This is the first study aimed at creating principles for chronic pain management in the TSCI population, in the developing context of the Western Cape of South Africa. A study published in 2012^33^ includes guidelines for neuropathic pain in South Africa. However, these guidelines are not specific for the TSCI population nor does it include nociceptive pain management. Our study yielded holistic assessment, management and monitoring principles for chronic pain (neuropathic and nociceptive) in the TSCI population. Chronic pain is multifaceted, defined as pain lasting longer than three months and influenced by a complex interaction of biopsychosocial factors.^34^ A holistic approach to chronic pain was recommended as early as 1977,^18,35^ with the biopsychosocial approach to chronic pain management aimed to address biological, physical, psychological and social ramifications of chronic pain in patients.^34^

In the present study, the first-line primary health care providers highlighted staff shortages as a challenge to interprofessional collaboration to holistically manage chronic pain in the TSCI population. This is consistent with other studies conducted in South Africa, where staff shortages are evident in developing contexts.^20,21^ Furthermore, challenges to holistic management of care in South Africa includes ineffective referral between interdisciplinary teams, such as technical complications and knowledge of referral patterns.^20,21^ In addition to the challenge of staff shortage in limiting holistic approaches of care, these staff shortages and high patient workload may also explain the 74% drop-out rate in relation to participation in round two of our Delphi Study. Health care providers in South Africa are burdened by a lack of resources, a lack of time, increased hours and a high patient to provider ratio increasing the risk of burnout amongst this population.^20,21,36^ The lack of psychologists and pain specialists at the primary health care level in the South African context is of great concern as the assessment and management provided by these professionals are part of the holistic approach to chronic pain management.^37,38^ Furthermore, the intensity of pain as well as pain-related disability has shown improvement through psychotherapeutic techniques, such as CBT, in the SCI population.^39-41^

Our Delphi study revealed that consensus could not be reached on the second to fourth-line pharmacological management of chronic pain in the TSCI population, in a developing context. There could be several reasons for this non-consensus, ranging from the lack of pain specialists at the primary health care level in the South African context, a lack of resources, such as staff training and skill support in pain management which is evident in the challenges faced by health care providers in relation to holistic care,^20,21^ the complexity of chronic pain, the issue of high prevalence rates of substance abuse in the TSCI population.^4^

### Implications for Clinical Practice

These principles (Figure 1) highlight the agreement by first-line primary health care providers that a holistic biopsychosocial approach to chronic pain assessment, management and monitoring should be prioritised, in the TSCI population, in the Western Cape of South Africa. Despite the challenges present in the South African context, health care providers should firstly be aware of the best available contextual evidence and, secondly, work towards incorporating these principles into the management of chronic pain at the primary health care level.

The lack of consensus regarding the second- to fourth-line pharmacological management of neuropathic pain draws attention to the need for the government, to make the appropriate drugs available at the primary health care level and for institutions to provide the necessary staff training for the improvement of chronic pain pharmacological management at the primary health care level.

### Limitations of the Study

Eighteen participants did not participate in the second round of the Delphi study despite numerous follow-up emails and in-person recruitment. A limitation of the study therefore is that a larger cohort of first-line primary health care providers may have resulted in a richer data set. Furthermore, the study did not include health care policymakers and pharmacy managers who may understand the economic and regulatory decisions for the lack of availability of certain drug treatment classes at specific levels of care. In addition, the inclusion of psychologists and pain specialists would provide further insights into the necessary care required to manage chronic pain in the TSCI population, however, these are not available at the primary health care level in the Western Cape region of South Africa.

## Conclusion

This study, the first of its kind in the developing context of the Western Cape of South Africa, provides agreed upon principles for chronic pain management in the TSCI population, particularly at the primary health care level. Future studies should assess which design principles are currently being implemented at the primary health care level and the factors associated with the implementation. Thereafter, studies should assess the impact of the implementation, which include the challenges and enablers.

## Data Availability

The data that support the findings of this study are available on request from the corresponding author, [TW]. The data are not publicly available due to containing information that could compromise the privacy of research participants.*
